# High tibial osteotomy versus unicompartmental knee arthroplasty for Kellgren–Lawrence grade 3–4 knee osteoarthritis in younger patients: comparable improvements in patient-reported outcomes, adjusted for osteoarthritis grade and sex

**DOI:** 10.1007/s00167-023-07526-5

**Published:** 2023-08-12

**Authors:** A. Hoorntje, Y. Pronk, J. M. Brinkman, R. C. I. van Geenen, R. J. van Heerwaarden

**Affiliations:** 1 Department of Orthopaedic Surgery and Sports Medicine, Amsterdam UMC Location University of Amsterdam, Meibergdreef 9, 1105 AZ Amsterdam, The Netherlands; 2 Amsterdam Movement Sciences, Program Musculoskeletal Health, Amsterdam, The Netherlands; 3 Research Department, Kliniek ViaSana, Mill, The Netherlands; 4 Department of Orthopaedic Surgery, Kliniek ViaSana, Mill, The Netherlands; 5 Department of Orthopaedic Surgery, Foundation for Orthopaedic Research Care and Education, Amphia Hospital, Breda, The Netherlands

**Keywords:** Unicompartmental knee arthroplasty, High tibial osteotomy, Osteoarthritis, Oxford knee score, Patient-reported outcome scores, Satisfaction

## Abstract

**Purpose:**

Previous studies comparing high tibial osteotomy (HTO) with unicompartmental knee arthroplasty (UKA) have seldom accounted for differing patient characteristics between both groups. This study compared patient-reported outcomes (PROs) of HTO and UKA patients, adjusted for preoperative PROs, osteoarthritis grade and sex.

**Methods:**

A retrospective study was performed analysing prospectively collected PROs, namely the Oxford Knee Score (OKS) and pain/satisfaction scores, collected preoperatively and at 6 months, 12 months and 24 months postoperatively. Consecutive medial opening-wedge HTOs and medial UKAs from 2016–2019, with a preoperative Kellgren–Lawrence grade ≥ 3, aged 50–60 years, were included. Linear mixed model analyses, with the OKS over time as the primary outcome, were used.

**Results:**

We included 84 HTO patients (mean age 55.0 ± 3.0, 79% male, mean BMI 27.8 ± 3.4, 75% Kellgren–Lawrence grade 3) and 130 UKA patients (mean age 55.7 ± 2.8, 47% male, mean BMI 28.7 ± 4.0, 36% Kellgren–Lawrence grade 3). Response rates were ≥ 87% at all time points. Corrected for preoperative PROs, Kellgren–Lawrence grade and sex, the HTO group had a 2.5 (95% CI 1.0–4.0) points lower OKS over time than the UKA group (*p* = 0.001). The Numeric Rating Scale scores (NRS; 0–10) for pain at rest and during activity were higher (*p* < 0.01) in the HTO group. The EQ-5D-descriptive system (*p* < 0.01), NRS satisfaction (*p* < 0.01), anchor function and pain scores (*p* < 0.01) were lower over time in the HTO group.

**Conclusion:**

UKA patients had better OKS scores, pain and satisfaction scores over time than HTO patients. However, the observed differences were below their established minimal clinically important differences. Therefore, from the patients’ perspective, HTO did not appear to be inferior to UKA under the indications outlined in this study.

**Level of evidence** Level IV.

## Introduction

For relatively young patients (50–60 years of age) with debilitating medial unicompartmental knee osteoarthritis (OA), high tibial osteotomy (HTO) and medial unicompartmental knee arthroplasty (UKA) are suitable surgical treatment options [26]. To date, more reviews and meta-analyses than randomized, prospective studies have been published on the topic of HTO versus UKA [2, 4, 6, 11, 14, 28, 34, 37]. Overall, UKA appears to result in better outcomes regarding pain relief and satisfaction, while range-of-motion seems higher after HTO.

Interestingly, previous studies have seldom elaborated on the effect of the preoperative indication, including preoperative OA grade, on the outcome of HTO or UKA [6]. Yet, valgus-producing HTO is most often advised for patients aged 40–60 years, with an extra-articular tibial varus deformity, who are non-smokers, with a body mass index (BMI) < 30 kg/m^2^ and mild-to-moderate medial compartment OA. Although, among others, the UK Knee Osteotomy Consensus Group recently stated that bone-on-bone OA should not be considered a strict contraindication for HTO [5, 26, 35]. In contrast, UKA is primarily indicated in patients aged > 60 years, without BMI restrictions, for treatment of anteromedial osteoarthritis (AMOA). For AMOA, the Oxford criteria apply and medial compartment bone-on-bone OA is a strict prerequisite [13, 30]. Performing UKA in patients without bone-on-bone medial OA results in worse patient-reported outcomes (PROs), and higher rates of reoperation and revision [12, 27, 29]. These criteria show that indications, including preoperative OA grade, for HTO and UKA only partly overlap. This could be the reason that only two randomized studies of suboptimal methodological quality have reported results for HTO versus UKA [4, 37]. These studies used outdated inclusion criteria, i.e. Ahlbäck OA grade I–III for both procedures, did not perform a power analysis, and included relatively small samples.

Thus, from a patients’ perspective it remains unclear if patients in the age range 50–60 years with moderate-to-severe medial OA benefit more from one of these procedures. Several reviews noted that future prospective, preferably randomized studies, are needed to define true differences between both procedures. To allow for a true comparison between HTO and UKA, one should compare patients who could have been indicated for both procedures based on their preoperative characteristics, including the preoperative radiographic OA severity. Also, other patient and surgeon factors, e.g. the wish to participate in high-impact activities, play a role in decision-making in younger, active patients.

Therefore, the aim of the present study was to retrospectively compare HTO and UKA on prospectively collected PROs during the first two years after surgery in comparable patients, based on preoperative characteristics including OA grade and sex. The hypothesis was that HTO would result in similar PROs compared to UKA in this selected population.

## Materials and methods

### Study design and patient selection

A single-centre retrospective study with prospectively collected data of consecutive non-randomized HTO and UKA patients operated on by dedicated knee surgeons between January 2016 and December 2019 in a high-volume clinic was performed. Guidelines of the Dutch Health Regulatory Agency state that patients with an American Society of Anesthesiologists (ASA) score > II and a BMI > 35 kg/m^2^ are not allowed to be operated on in the present study’s clinic. Therefore, all patients in the present study had an ASA score of I–II and a BMI < 35 kg/m^2^.

Eligible patients had medial OA Kellgren–Lawrence grade (KL grade) ≥ 3, were aged between 50 and 60 years, provided a signed informed consent, and underwent HTO or UKA. Patients who were operated on both knees were included in this prospective database if their second surgery was > 6 months after the first surgery (*n* = 6). Exclusion criteria included OA in more than one compartment, a preoperative radiograph of insufficient quality, and double level (i.e. tibia and femur) osteotomies. The KL grade was determined by two independent researchers on regular preoperative standing AP knee radiographs and on stress radiographs if available [22]. KL grade 4 was assigned in cases with medial bone-on-bone. Discrepancies were resolved by discussion.

### Surgical technique

For HTO, a medial opening-wedge technique with biplanar osteotomy was performed [15]. Plate fixation in all patients was performed with angular stable plates (TomoFix, Synthes GmbH, Switzerland). No grafts were used. Postoperatively, immediate range-of-motion exercises and muscle strengthening were started with guidance from a physical therapist. All patients were allowed full weight-bearing from 2 weeks postoperatively.

For UKA, standard surgical technique was performed as described by the manufacturer. Either a fixed bearing implant, the Physica ZUK UKA (LIMA Corporate UD, Italy) or a mobile bearing implant, the cementless Phase III Oxford UKA (ZimmerBiomet Ltd., Bridgend, UK) was used. All patients were allowed direct full weight-bearing postoperatively.

**Fig. 1:**
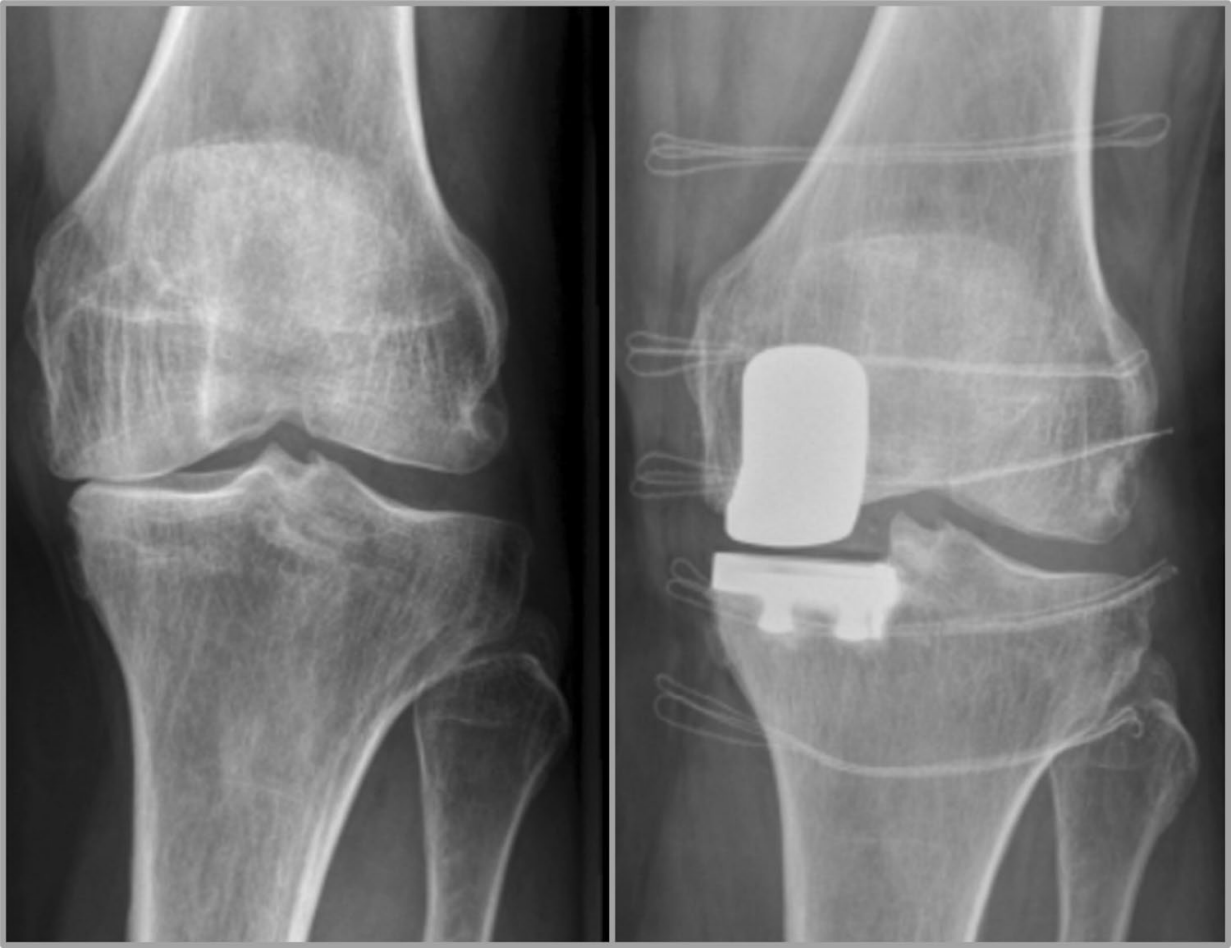
Clinical case of female, 58 years, left knee; Kellgren–Lawrence grade 4; medial UKA chosen by patient and surgeon (**A** preoperative X-ray, **B** postoperative X-ray)

**Fig. 2:**
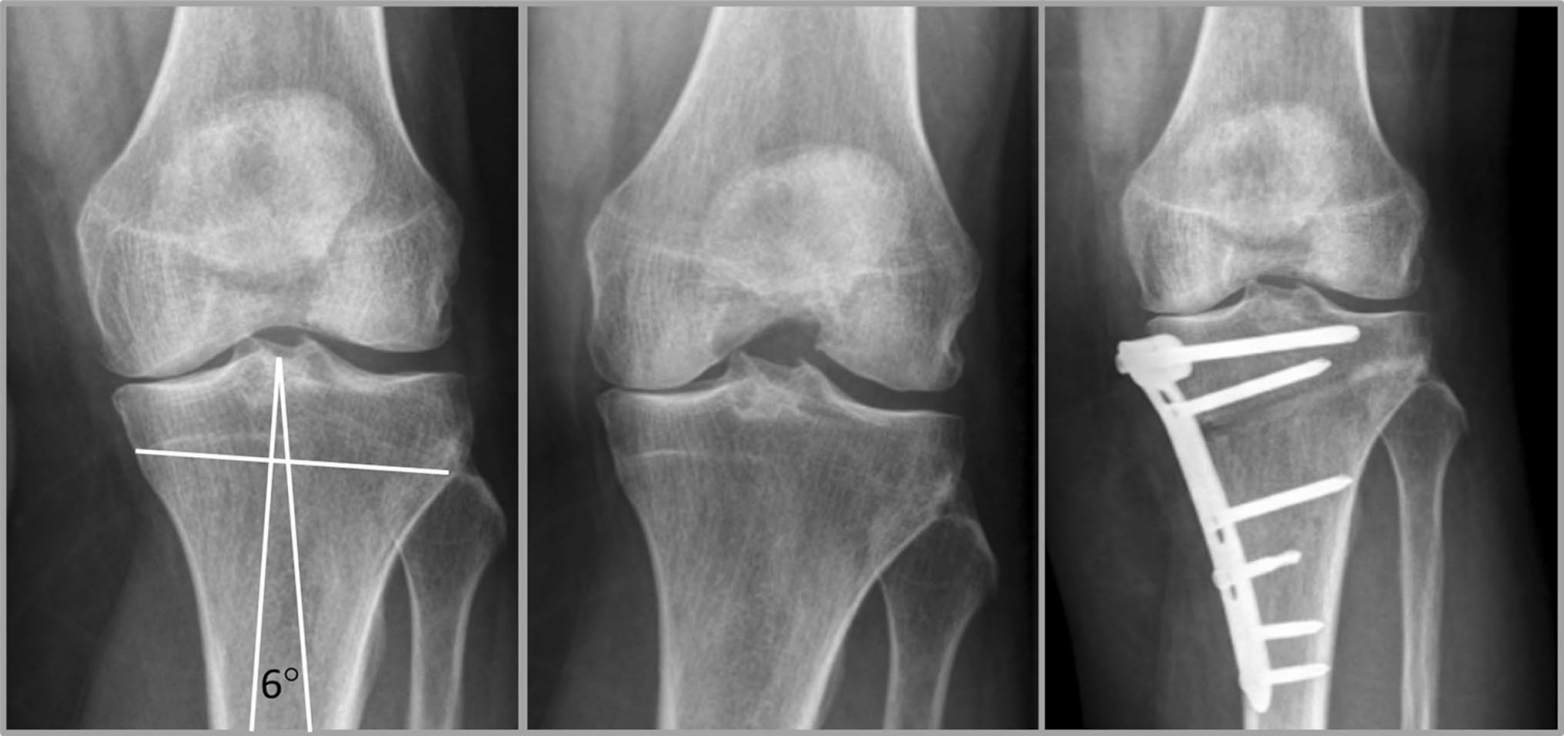
Clinical case of female, 54 years, left knee; Kellgren–Lawrence grade 3–4, tibial bone varus angle (TBVA) shows extra-articular deformity; HTO preferred by patient because of high physical demands (**A** preoperative X-ray + TBVA measurement [3], **B** preoperative stress X-ray, **C** postoperative X-ray)

The preferred treatment option was selected based on surgeon and patient preference (Table 1; Figs. 1 and 2).

**Table 1:** Factors influencing treatment selection in younger, active patients with medial knee osteoarthritis

	HTO	UKA
*Surgeon factors*		
Radiological: KL grade 3	X	
Radiological: KL grade 4		X
TBVA increased (extra-articular varus deformity) [3]	X	
TBVA normal (intra-articular varus deformity) [3]		X
Patient compliant for treatment and rehabilitation	X	
Previous treatments/complications		
*Patient factors*		
Strong preference for HTO or UKA	X	X
Active lifestyle with high joint loading demands	X	

X represents the preferred treatment option, but treatment selection was made based on shared decision-making

*HTO* high tibial osteotomy, *UKA* unicompartmental knee replacement, *KL* Kellgren–Lawrence, *TBVA* tibial bone varus angle

### Outcomes and measures

The primary outcome was the change in Oxford Knee Score (OKS) over time (from preoperative to 24 months postoperative).

Secondary outcomes included pain, quality of life, patient satisfaction and anchor scores for pain, function and recovery. Pain at rest and during activity were measured using a numeric rating scale (NRS; 0 no pain, 10 worst imaginable pain). Quality of life was measured by the 5-level version of EuroQol 5 Dimensions (EQ-5D-5L) [40]. A NRS to measure patient satisfaction was used (0–10, 0 dissatisfied to 10 very satisfied). The question asked was ‘*How satisfied are you (in general) with the results of your knee surgery?’*. Also, anchor questions were asked for pain (1–7, 1 very much deteriorated to 7 very much improved), function (1–7, 1 very much deteriorated to 7 very much improved) and recovery measured using the general perceived recovery questionnaire (GPR; 0–6, 0 worse than ever to 6 fully recovered) [20, 32].

PROs were routinely collected using an online, automated system (OnlinePROMs, Interactive Studios, The Netherlands). Patients completed questionnaires preoperatively and at 6 months, 12 months and 24 months postoperatively. A maximum of two reminders were sent to complete the questionnaires. The treating orthopaedic surgeons did not have direct access to the PROs during follow-up visits.

Patient’s age at surgery (years), BMI (kg/m^2^), sex (male or female), ASA score (I or II), side of surgery (left or right), patient-specific instrumentation (yes or no), date of surgery, complications and reoperations including plate removal and date of reoperation were collected from the electronic patient file.

### Ethical approval

All patients provided written informed consent preoperatively, allowing the use of their anonymized data for future research. Therefore, the local ethical committee declared that they did not require formal ethical approval for studies that use these anonymized data (IRB number: 2022–02).

### Statistical analysis

Statistical analysis was performed using IBM SPSS Statistics version 28.0 (IBM Corp, Armonk, New York). Results were reported as mean and standard deviation (mean ± SD), median and interquartile range (median (IQR)), and numbers with percentage (n (%)). To compare patient characteristics and preoperative PROs between the HTO group and the UKA group, continuous variables were first checked for normal distributions. Depending on normality of the data, independent *t*-tests or Mann–Whitney U tests were performed. For categorical variables, the Chi-square tests or Fisher’s exact tests were performed. Mixed linear models were used to analyse the overall decrease or increase for continuous outcome data. The linear models included the within-subject or group variable (HTO versus UKA) and included patient characteristics that differed between both groups as factors in the model. An alpha of 0.05 was considered statistically significant.

The primary outcome was the OKS over time. To estimate the population mean of the OKS, data from the Dutch Arthroplasty Register (LROI) were used. The mean 1-year OKS for 37,388 TKA patients was 38.6 (95% CI 38.5–38.7). The randomized controlled TOPKAT trial, comparing TKA and UKA, found no difference in OKS at five years between both procedures [1]. Therefore, this population mean for UKA patients was used in the present study. Next, Floerkemeijer et al. reported a median OKS of 43 points (range 8–48 points) after a mean follow-up of 3.6 years in 386 HTO patients [9]. The calculated sample size was based on the abovementioned mean OKS and the known minimal clinical important difference (MCID) of the OKS in knee arthroplasty patients, namely 5.0 points [7]. To demonstrate non-inferiority of HTO compared to UKA, a total of 50 patients were needed in each group. Adjusted for a 10% loss-to-follow-up, at least 55 patients needed to be included in each group.

## Results

In total 84 patients were included in the HTO group and 130 patients in the UKA group. In the HTO group, more male patients were included (66 (79%)) compared to the UKA group (61 (47%); *p* < 0.001). Also, the majority of HTO patients had a preoperative KL grade 3 (63 (75%)) while the majority of UKA patients had a preoperative KL grade 4 (83 (64%); *p* < 0.001). The other patient characteristics did not significantly differ between the groups (Table 2).

**Table 2:** Patient characteristics for the HTO group and UKA group

	UKA (*n* = 130)	HTO (*n* = 84)	*p*-value
Age at surgery (years), mean ± SD	55.7 ± 2.8	55.0 ± 3.0	0.086^#^
BMI (kg/m^2^), mean ± SD	28.7 ± 4.0	27.8 ± 3.4	0.094^#^
Sex (male), *n* (%)	61 (47%)	66 (79%)	< 0.001*
Side (right), *n* (%)	68 (52%)	43 (51%)	0.873*
ASA classification, *n* (%)			0.273*
I	77 (59%)	56 (67%)	
II	53 (41%)	28 (33%)	
KL grade, *n* (%)			< 0.001*
III	47 (36%)	63 (75%)	
IV	83 (64%)	21 (25%)	
PSI (yes), *n* (%)	96 (74%)	–	–
Plate removal (yes), *n* (%)	–	61 (73%)	–
Timing of plate removal (months from index surgery), median (IQR)	–	14.1 (11.5–7.9)	–
Complication, *n* (%)			–
Deep infection	1 (1%)	3 (4%)	
Fixation failure	–	1 (1%)	
Non-union (revision with allograft)	–	2 (2%)	
Re-arthroscopy	3 (2%)	1 (1%)	
GI bleeding	1 (1%)	–	
Infection after plate removal	–	2 (2%)	
Revision to TKA	1 (1%)	3 (4%)	

Bold indicates a *p*-value of <0.05

*ASA* American Society of Anesthesiologists, *BMI* body mass index, *GI* gastro-intestinal, *HTO* high tibial osteotomy, *KL *grade: Kellgren–Lawrence grade, *n* number, *PSI* patient-specific instrumentation, *TKA* total knee arthroplasty, *UKA* unicompartmental knee arthroplasty

*Chi-square test, ^#^ Independent samples *t*-test. Significance (values in bold) was assumed at *p* < 0.05

Response rates for the HTO group were 100% (*n* = 84) preoperatively, 90% (*n* = 76) at 6 months, 87% (*n* = 73) at 12 months and 87% (*n* = 73) at 24 months postoperatively. Response rates for the UKA group were 100% (*n* = 130) preoperatively, 90% (*n* = 117) at 6 months, 95% (*n* = 123) at 12 months and 91% (*n* = 118) at 24 months postoperatively. No significant differences in response rates between both groups were found (*p* > 0.05). Three HTO patients (4%) and one UKA patient (1%) were converted to TKA during follow-up (Table 2).

The preoperative OKS was significantly higher in the HTO group (26.6 ± 8.0) than in the UKA group, (23.9 ± 6.9; *p* = 0.010). Also, the preoperative EQ-5D descriptive system score was significantly higher in the HTO group (0.663, IQR 0.547–0.663) than in the UKA group (0.663 IQR 0.663–0.744; *p* = 0.001). The other preoperative PROs did not significantly differ between both groups (Table 3).

**Table 3:** Patient-reported outcomes from preoperative to two years postoperative for the HTO group and UKA group

	UKA (*n* = 130)	HTO (*n* = 84)	*p*-value
*Oxford Knee Score, mean ± SD or IQR*			
Preoperative	23.9 ± 6.9	26.6 ± 8.0	0.010*
6 months	41.0 (36.0–45.0)	35.5 (29.0–43.0)	** < 0.001**^**#**^
12 months	44.0 (39.0–46.0)	42.0 (33.0–46.0)	**0.033**^**#**^
24 months	44.0 (39.0–47.0)	44.0 (36.0–46.0)	0.502
*NRS pain at rest, median (IQR)*			
Preoperative	6 (5–7)	6 (4–7)	0.338^#^
6 months	1 (0–2)	2 (0–4)	**0.002**^**#**^
12 months	0 (0–1)	1 (0–4)	**0.003**^**#**^
24 months	0 (0–2)	0 (0–2)	0.406^#^
*NRS pain during activity, median (IQR)*			
Preoperative	8 (7–9)	8 (7–8)	0.079^#^
6 months	2 (0–4)	4 (2–7)	**0.002**
12 months	1 (0–2)	3 (1–5)	** < 0.001**^**#**^
24 months	1 (0–3)	2 (0–3)	**0.021**^**#**^
*EQ-5D descriptive system, median (IQR)*			
Preoperative	0.663 (0.547–0.663)	0.663 (0.663–0.744)	**0.001**^**#**^
6 months	0.840 (0.743–0.947)	0.663 (0.663–0.818)	** < 0.001**^**#**^
12 months	0.947 (0.786–0.947)	0.766 (0.663 = 0.947)	** < 0.001**^**#**^
24 months	0.947 (0.771–0.947)	0.947 (0.786–0.947)	0.783^#^
*EQ VAS, median (IQR)*			
Preoperative	77.0 (65.0–89.3)	80.0 (70.0–90.0)	0.103^#^
6 months	82.5 (75.0–90.0)	78.5 (64.3–86.8)	**0.011**^**#**^
12 months	84.5 (75.0–93.0)	80.0 (70.0–90.5)	0.096^#^
24 months	82.0 (72.0–91.0)	80.0 (73.3–90.8)	0.897^#^
*NRS satisfaction, median (IQR)*			
months	9 (8–10)	7 (6–8)	** < 0.001**^**#**^
12 months	9 (8–10)	8 (7–9)	**0.001**^**#**^
24 months	9 (8–10)	9 (7–10)	**0.026**
*Anchor pain score, median (IQR)*			
6 months	6 (6–7)	6 (5–6)	** < 0.001**^**#**^
12 months	7 (6–7)	6 (5–7)	** < 0.001**^**#**^
24 months	7 (6–7)	6 (6–7)	**0.018**^**#**^
*Anchor function score, median (IQR)*			
6 months	6 (6–7)	5 (4–6)	** < 0.001**^**#**^
12 months	6 (6–7)	6 (5–7)	** < 0.001**^**#**^
24 months	7 (6–7)	6 (6–7)	**0.044**^**#**^
*GPR, median (IQR)*			
6 months	5 (5–6)	5 (4–5)	** < 0.001**^**#**^
12 months	5 (5–6)	5 (4–5)	** < 0.001**^**#**^
24 months	6 (5–6)	5 (5–6)	**0.002**^**#**^

Bold indicates a *p*-value of <0.05

*GPR* general perceived recovery, *HTO* high tibial osteotomy, *NRS* numeric rating scale (0–10), *OKS* Oxford Knee Score, *UKA* unicompartmental knee arthroplasty, *EQ-VAS* EuroQol visual analogue scale

*Independent samples *t*-test for preoperative values, ^#^ Mann–Whitney U test for preoperative values

*p* < 0.05 was considered significant (values in bold)

**Fig. 3:**
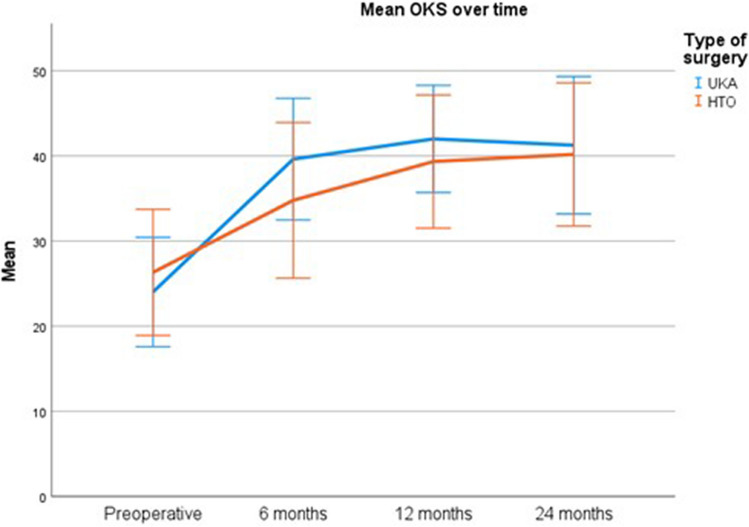
Mean OKS over time for the HTO group and UKA group. Error bars represent standard deviation

The OKS over time was 2.5 points (95% CI 1.0–4.0) lower for the HTO group compared to the UKA group (*p* < 0.001), corrected for preoperative KL grade and sex (Fig. 3). The same model showed that patients with a KL grade 3 had a 1.6 (95% CI − 3.0–− 0.2) points lower OKS over time compared to KL grade 4 (*p* = 0.03), and males had a 2.3 points (95% CI 0.9–3.7) higher OKS over time than females (*p* < 0.001) (Table 4).

**Table 4:** The effect of type of surgery corrected for preoperative KL grade and sex on the primary outcome OKS over time

Primary outcome	Effect	Reference	B	95% CI	*p*-value
OKS	Type of surgery	HTO	**2.5**	**1.0–4.0**	**0.001**
	KL grade	KL grade 4	**−** **1.6**	**−** **3.0 to −** **0.2**	**0.027**
	Sex	Female	**2.3**	**0.9–3.7**	**0.001**

Bold indicates a *p*-value of <0.05

*HTO* high tibial osteotomy, *KL* Kellgren–Lawrence, *OKS* Oxford Knee Score

*p* < 0.05 was considered significant (values in bold)

For the secondary outcomes, the NRS pain in rest score and NRS pain during activity score over time were higher in the THO group that the UKA group. The EQ-5D descriptive system score, NRS satisfaction score and anchor scores for pain, function and GPR were lower over time in the HTO group (Table 5). Patients with KL grade 3 had lower EQ-5D scores over time compared to KL grade 4, and males had a higher EQ-5D score over time than females (Table 5).

**Table 5:** The effect of type of surgery corrected for preoperative KL grade and sex on the secondary outcomes over time

Secondary outcome	Effect	Reference	B	95% CI	*p*-value
NRS pain in rest score	Type of surgery	HTO	− **0.7**	− **1.1** to − **0.4**	** < 0.001**
	KL grade	KL grade 4	0.2	− 0.1–0.6	0.220
	Sex	Female	− 0.1	− 0.5–0.2	0.425
NRS pain during activity score	Type of surgery	HTO	− **1.2**	− **1.7** to − **0.8**	** < 0.001**
	KL grade	KL grade 4	0.3	− 0.1–0.7	0.114
	Sex	Female	− 0.4	− 0.8–0.03	0.069
EQ-5D descriptive system score	Type of surgery	HTO	**0.051**	**0.020**–**0.083**	**0.001**
	KL grade	KL grade 4	− **0.030**	− **0.060** to − **0.001**	**0.044**
	Sex	Female	**0.036**	**0.007**–**0.065**	**0.015**
EQ VAS score	Type of surgery	HTO	1.2	− 1.5–3.8	0.403
	KL grade	KL grade 4	− 1.0	− 3.5–1.5	0.442
	Sex	Female	0.7	− 1.8–3.1	0.609
NRS satisfaction score	Type of surgery	HTO	**1.0**	**0.6**–**1.3**	** < 0.001**
	KL grade	KL grade 4	− 0.2	− 0.5–0.1	0.193
	Sex	Female	0.3	− 0.02–0.6	0.063
Anchor pain score	Type of surgery	HTO	**0.6**	**0.4**–**0.8**	** < 0.001**
	KL grade	KL grade 4	− 0.1	− 0.3–0.1	0.343
	Sex	Female	0.1	− 0.1–0.2	0.518
Anchor function score	Type of surgery	HTO	**0.7**	**0.5**–**0.9**	** < 0.001**
	KL grade	KL grade 4	0.002	− 0.2–0.2	0.984
	Sex	Female	0.1	− 0.1–0.3	0.150
GPR score	Type of surgery	HTO	**0.6**	**0.4**–**0.8**	** < 0.001**
	KL grade	KL grade 4	− 0.1	− 0.2–0.1	0.493
	Sex	Female	0.1	− 0.1–0.2	0.346

Bold indicates a *p*-value of <0.05

*GPR* general perceived recovery, *HTO* high tibial osteotomy, *KL* Kellgren–Lawrence, *NRS* numeric rating scale, *OKS* Oxford Knee Score, *EQ-VAS* EuroQol visual analogue scale

*p* < 0.05 was considered significant (values in bold)

## Discussion

Our most important findings were slightly better results over time for the UKA group compared to the HTO group in terms of function scores (OKS), scores for pain in rest and during activity, quality of life scores, patient satisfaction scores and anchor scores for pain, function and GPR during the first 24 months after surgery.

Yet, the issue of statistical significance versus clinical relevance is more difficult to address. The MCID for the OKS is 5.0 points [7]. For VAS pain and satisfaction scores, the MCID for joint arthroplasty procedures is assumed to be around 16 points on a 100 points VAS [8, 21, 24], which could be translated to 1.6 points on a 10 point NRS. For the EQ-5D, the MCID is assumed to be around 0.074 [38]. Thus, differences in PROs between HTO and UKA in the present study were below their MCIDs. Regarding the higher preoperative OKS and EQ-5D for the HTO group, this was accounted for in the analysis by analysing the improvement over time for the included PROs as the primary outcome measure.

Previous studies have seldom accounted for the effect of OA grade on PROs after HTO versus UKA. The present study only included KL grade 3 and 4, and adjusted for the difference in baseline OA grade in the linear mixed models assessing PROs. In comparison, Jacquet et al. did not statistically adjust for OA grade, but only included KL grade 2–3 for their comparison, in patients who previously participated in high-impact sports [17]. After 24 months, mean UCLA Activity Scores, Knee injury and Osteoarthritis Outcome Score (KOOS) Sports-subscale scores, and Knee Society Scores were significantly higher in the HTO group. Furthermore, 62% of HTO patients practised impact sports after 24 months, compared to 28% in the UKA group [17]. Recently, Sakai et al. compared HTO and TKA, correcting for age, OA grade and preoperative KOOS in patients > 60 years of age [33]. The authors found no difference in short-term pain relief and improvements measured by KOOS subscales. In addition, a meta-analysis found higher pre- and post-operative activity levels for HTO compared to UKA, while UKA patients showed greater pre- to post-operative improvements [2]. Pooled mean postoperative OKS was 36.7 points for the HTO group and 35.0 points for the UKA group [2], compared to a median OKS of 44 at 24 months in both groups in the present study. Unfortunately, in their meta-analysis the authors did not adjust for preoperative OA grade, limiting the applicability of their results. Thus, limited data show that HTO performs as well or better than UKA in terms of physical activity PROs when adjusting for preoperative OA grade.

Systematic reviews investigating functional outcomes consistently showed that UKA provides better postoperative pain relief and lower revision rates than HTO [6, 14, 31, 34]. Also, postoperative recovery is generally quicker after UKA than HTO [23]. For postoperative complications, several reviews report lower rates after UKA [6, 14, 34], although a 2022 South Korean registry study including 21,194 UKAs and 49,270 HTOs found higher rates of deep venous thrombosis and surgical site infection after UKA [25]. Complications found in both groups in the present study are comparable to those reported in other studies. Although the number of complications found in the present study were lower in UKA versus HTO, several complications in the HTO group occurred in a single patient. Additionally, systematic reviews also consistently show better range-of-motion after HTO than UKA [6, 10, 31, 36]. For postoperative walking velocity, no clear advantage for either procedure was found [10, 14]. Lastly, another reason why some authors prefer HTO over UKA in young, active patients, is the risk of early UKA revision. Registry studies show a significantly higher revision risk for patients < 55 years of age compared to patients > 70 years of age (9% versus 3–4%), and of > 20.000 German UKA patients, one in five patients < 55 years of age underwent revision within five years [18, 41]. Overall, return to physical activities, including sports and work, is good to excellent after both procedures, with higher participation in work and high-impact sports after HTO [2, 16, 17, 39].

Clearly, using correct indications is important to obtain satisfactory results. Modern-day HTO should be primarily offered to relatively young and non-obese patients with mild-to-moderate medial compartment OA, although no strict restrictions for BMI and OA grade exist [26]. Most importantly, an extra-articular tibial varus malalignment must be present that can be corrected while respecting joint-line obliquity [5, 26]. The tibial bone varus angle (TBVA) is one of the decisive factors in preoperative decision-making for UKA vs. HTO, with a TBVA > 5 degrees being predictive of successful HTO [3]. UKA is a proven alternative but must be reserved for patients with medial bone-on-bone OA to guarantee optimal functional outcomes and prosthesis survival [13, 26]. Finally, performing HTO for KL grade 4 may result in higher rates of dissatisfaction and failure [19, 23], although we did not find this association in the present study. On the other hand, young age and severe varus deformity were associated with dissatisfaction after UKA [23]. Therefore, the orthopaedic surgeon should carefully consider the available evidence and the limited overlap in UKA and HTO indications, and discuss this with the patient preoperatively.

### Limitations

A limitation is the fact that preoperative stress radiographs were not available for all patients. Consequently, patients who were graded as KL grade 3 may have had a medial compartment KL grade 4 on stress radiographs. Also, external validity might be hampered by the fact that our clinic is a high-volume, dedicated UKA and HTO clinic. For the UKA group, two implant designs were used during the study period, although no difference in PROs was found between these two implant designs [32]. The follow-up of 24 months for both groups may represent another limitation, and follow-up data at 5 and 10 years would be interesting to compare. Next, full weight-bearing protocols differed between both groups. Lastly, selection bias was likely present in the present study, since data collection was prospective, but we did not perform a randomized study.

## Conclusion

Younger (50–60 years) patients had better function (OKS), pain and satisfaction scores over time after UKA than HTO, adjusted for preoperative PROs, OA grade and sex. Yet, the observed differences were below their established minimal clinically important differences. Both HTO and UKA are suitable treatment options that will likely lead to satisfactory PROs, although overlap in indications is very limited.

## Data Availability

No data availability statement is available. Authors may contact the first author for questions regarding usage of data from the present study.
